# Improving motion management in radiation therapy: findings from a workshop and survey in Australia and New Zealand

**DOI:** 10.1007/s13246-024-01405-0

**Published:** 2024-05-28

**Authors:** Elizabeth Claridge Mackonis, Rachel Stensmyr, Rachel Poldy, Paul White, Zoë Moutrie, Tina Gorjiara, Erin Seymour, Tania Erven, Nicholas Hardcastle, Annette Haworth

**Affiliations:** 1 University of Sydney, Camperdown, Australia; 2 Chris O’Brien Lifehouse, Camperdown, Australia; 3 Western Sydney LHD, Westmead, Australia; 4 Canberra Region Cancer Centre, Canberra, Australia; 5 South Eastern Sydney LHD, Randwick, Australia; 6 South Western Sydney Cancer Services, Sydney, NSW Australia; 7 Ingham Institute for Applied Medical Research, Sydney, Australia; 8 South Western Sydney Clinical School, University of NSW, Liverpool, NSW Australia; 9 Icon Cancer Care, Bella Vista, Australia; 10 Central Coast LHD, Gosford, Australia; 11 Peter MacCallum Cancer Centres, Melbourne, Australia; 12 Institute of Medical Physics, University of Sydney, Camperdown, Australia

**Keywords:** Motion management, Australia, New Zealand, Australasia, Workshop, Survey

## Abstract

Motion management has become an integral part of radiation therapy. Multiple approaches to motion management have been reported in the literature. To allow the sharing of experiences on current practice and emerging technology, the University of Sydney and the New South Wales/Australian Capital Territory branch of the Australasian College of Physical Scientists and Engineers in Medicine (ACPSEM) held a two-day motion management workshop. To inform the workshop program, participants were invited to complete a survey prior to the workshop on current use of motion management techniques and their opinion on the effectiveness of each approach. A post-workshop survey was also conducted, designed to capture changes in opinion as a result of workshop participation. The online workshop was the most well attended ever hosted by the ACPSEM, with over 300 participants and a response to the pre-workshop survey was received from at least 60% of the radiation therapy centres in Australia and New Zealand. Motion management is extensively used in the region with use of deep inspiration breath-hold (DIBH) reported by 98% of centres for left-sided breast treatments and 91% for at least some right-sided breast treatments. Surface guided radiation therapy (SGRT) was the most popular session at the workshop and survey results showed that the use of SGRT is likely to increase. The workshop provided an excellent opportunity for the exchange of knowledge and experience, with most survey respondents indicating that their participation would lead to improvements in the quality of delivery of treatments at their centres.

**Supplementary Information:**

The online version contains supplementary material available at 10.1007/s13246-024-01405-0.

## Introduction

Strategies to control, limit or correct for the motion of targets and organs-at-risk (OAR) during radiation therapy can be broadly considered as motion management. While motion management is often focused on respiratory motion, it may also be applied to digestive, cardiac and other physiological motion [1].

Motion management in the form of deep inspiration breath-hold (DIBH) has been shown to reduce cardiac toxicity for breast radiation therapy patients [2]. As a result, this technique is recommended for left-sided breast radiation therapy patients and is widely utilised [3, 4].

Motion management using techniques such as gating and breath-hold is used in other thoracic and upper abdominal treatments to minimise tumour motion and associated target margins, to spare adjacent OARs. This is particularly pertinent in stereotactic ablative body radiotherapy (SBRT), where escalated target doses are used. The use of SBRT is increasing in Australia [5] and it is recognized as an emerging standard of care for a range of early stage or metastatic cancers in the thorax and upper abdomen [6–8]. Management of respiratory motion has come to be recognized as a key requirement for these treatments [9, 10].

With the uptake of various methods of motion management increasing, the NSW/ACT branch of the ACPSEM collaborated with the University of Sydney to host a two-day workshop on motion management. The workshop was designed to identify and communicate emerging trends, and to facilitate the sharing of practical experience. To address these aims, the workshop invited speakers to present an overview of their experience in using a range of motion management equipment and techniques, including the advantages and disadvantages. In addition, radiation therapy centres were surveyed to identify equipment and methods currently in use and opinions on their effectiveness. Following the workshop, participants were again surveyed to identify any changes in opinion resulting from the information and panel discussions presented.

Here we present an executive summary of the motion management techniques that were in common use at the time of the workshop and the opinions of the participants regarding the practical advantages and limitations of each. This summary is intended to promote further discussion across the community.

## Method

The workshop was hosted virtually on the 27th and 28th of July 2022, in two 5 h sessions. This format was chosen to facilitate participation from the ACPSEM membership around clinical duties, and mitigate difficulties associated with travel required for in-person meetings. Invitations to the workshop were sent via the ACPSEM email list, the Australian Society of Medical Imaging and Radiation Therapy (ASMIRT) NSW/ACT email list and the University of Sydney, Institute of Medical Physics email list, and the event was advertised on the ACPSEM website, on the Better Healthcare Technology Foundation website, on social media and in the ACPSEM newsletter.

The program was devised by an organizing committee of experienced radiation oncology medical physicists with topics covered in Table 1. To ensure broad applicability, invited speakers with experience using a diverse range of motion management equipment and techniques were selected from radiation therapy centres around Australia. They were encouraged to focus on the practicalities and limitations of specific motion management techniques, as well as the underlying principles. The program included dedicated time for discussion and facilitated questions using the online chat.

**Table 1:** Summary of the topics covered in the workshop

The current use of motion management
Motion management aspects of simulation and planning
Surface guided monitoring
Imaging and monitoring on treatment
Practical experience of radiation oncology centres in NSW/ACT
Motion management for clinical trial
Motion management into the future

Three online surveys were developed using Survey Monkey (www.surveymonkey.com) to accompany the workshop: a current practice survey (Survey A), an initial opinions survey (Survey B), and a post-workshop opinions survey (Survey C). Survey A was targeted at clinical departments at an organizational level, focused on the utilisation of motion management and availability of equipment for motion management. In contrast, Surveys B and C were aimed at gaining the opinions of individuals prior to and following the workshop to look at differences and determine if the workshop changed individual’s opinions on different topics. The survey questions were developed collaboratively by the organizing committee and are shown in Appendix 1. Surveys A and B were sent with the invitation to register for the workshop and Survey C was sent to all registered participants following the workshop.

All survey responses were collected anonymously. Respondents were asked to nominate a single representative from their centre to complete Survey A, and to indicate the name of their centre, to avoid duplication of data. This information was not used in the analysis. The surveys utilised logic to remove or skip irrelevant questions based on previous responses, thereby minimizing the time for respondents, and preventing the collection of unneeded data. Due to this logic filtering, some questions only received a small number of responses. To ensure anonymity, only questions with 5 or greater responses are reported. Analysis was performed using Microsoft Excel (Microsoft Corporation. (2018) Version 2301).

## Results

The online workshop attracted over 300 participants (the most well attended workshop ever hosted by the ACPSEM at the time), demonstrating a high level of interest in motion management. While time was allocated for discussion and questions, it was often insufficient to answer all the questions from attendees.

The results from Survey C indicated 54% of survey respondents felt that the workshop did change their opinions about motion management and 83% felt that, as a result of attending the workshop, changes could be made at their centre to improve treatment quality (Survey C, 24 responses). At the workshop conclusion, the session on surface guided radiation therapy (SGRT) was rated the most valuable (Survey C, 24 responses).

Several questions were repeated in both Survey B and Survey C in order to measure the effect of the workshop on opinions. Only small differences were seen in the responses to these questions, so they did not provide quantitative evidence that the workshop had an impact on participants’ opinions.

High response rates were received to both Survey A and B. Survey A received 66 responses, each representing a separate radiation therapy centre. Of these, 3 respondents indicated that they do not currently work in a radiation therapy centre and 4 people choose not to list their workplace. All workplaces were unique locations and from Australia or New Zealand. This represents a response rate of at least 60% of the 99 radiation therapy centres in Australia and New Zealand [11, 12]. For Survey B, 97 responses were received with the largest number of responses from radiation oncology medical physicists (83%) with 14% from radiation therapists and 3% from radiation oncologists. For Survey C, 33 responses were received, all from radiation oncology medical physicists.

### Deep inspiration breath-hold and surface guided radiation therapy

**Fig. 1:**
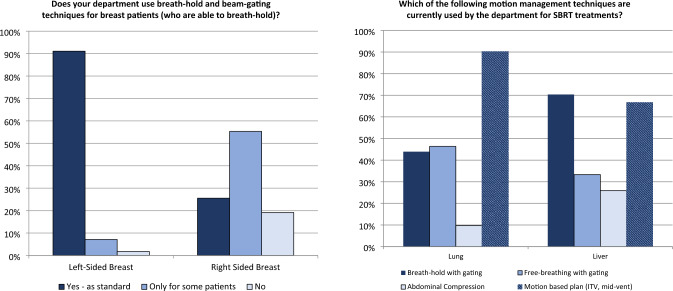
(Left) Application of breath-hold and beam-gating for breast patients by laterality (Survey A, 56 responses). (Right) Methods of motion management by site for SBRT (Survey A, 42 responses), responses only from centres treating SBRT for these sites

Motion management is most commonly used for breast treatments (Table 2, Survey A) with 98% of centres using breath-hold and beam gating treatment delivery for left-sided breast treatments and 91% considering it as standard practice (Fig. 1). This technique is also commonly used for right-sided breast treatments with 91% of centres using this form of motion management for at least some right-sided breast radiation therapy patients. These results are higher than those reported in international studies such at the POP-ART RT survey conducted by ESTRO in 2019 [13] where 63% of respondents indicated that deep inspiration breath-hold (DIBH) was used for breast cancer at their centre, and the AAPM Task Group 324 survey in 2020 [14] where 84% of respondents indicated the use DIBH for left-sided breast radiation therapy.

With the expansion of DIBH, many questions at the workshop were focused on the practicalities of providing this service, such as infection risks and consumable costs for a range of technologies. These concerns have led some centres to move from using semi-invasive active breathing control systems to SGRT systems for breath-hold gating. This shift in practice was also evident in survey A that showed 25% of centres are using SGRT for motion management, with 40% indicating that such an approach was currently being procured, implemented, or was being considered for the future (Survey A, 48 responses). Compared to the recent SGRT survey results from Batista et al. [15], an international survey, where 49% of institutions were using SGRT clinically, the lower usage locally shows the potential for future growth.

The panelists at the SGRT session agreed that surface guidance is unlikely to replace x-ray based positional verification imaging. However, by allowing better initial patient positioning and on-going monitoring, it has the potential to reduce the number of x-ray images taken and to reduce setup times once the staff become familiar with the system. Panelists discussed the steep learning curve and the need to ensure radiation therapist training and credentialing is incorporated into implementation planning to ensure efficient and accurate patient treatment.

### Patient focused motion management

Many of the workshop presentations demonstrated the clear benefits of motion management for patients resulting from the reduced dose to OARs, but discussion also indicated that it is important to consider the needs of the individual patient to ensure the motion management approach used is appropriate [16]. For example, some patients feel more comfortable in voluntary breath-hold compared with active breath-hold, but if an SGRT based system is used to monitor the breath-hold, this may lead to different issues, as some patients may not wish to be uncovered for treatment. Another comment regarded the sensitivity around marketing of motion management, since patients may feel disappointed, or concerned about the quality of their treatment if they are assessed to be unsuitable for breath-hold or another motion management technique.

When deciding on a motion management strategy, 87% of centres stated that they perform some form of patient screening to assess suitability. Breathing assessments were performed at simulation at 83% of all centres (Survey A, 46 responses), motion assessments were performed at simulation at 94% of all centres (Survey A, 46 responses) and patient breathing assessments were performed prior to simulation at 42% of all centres (Survey A, 40 responses). Screening of patients was considered to be important or very important by 96% of respondents (Survey B, 57 responses). Breathing assessments are most commonly performed using the Varian RPM (Varian Medical Systems, Palo Alto, CA, USA) system (Survey A, 67% of 43 responses) and target motion assessments are most often based on the treatment planning CT images (Survey A, 83% of 40 responses).

During the workshop, presenters showed tools used for determining the appropriate motion management technique for each patient, similar to the decision tree shown in a recent publication from a large Australian cancer centre [17]. For this institution, abdominal compression is only selected if exhale breath-hold and free-breathing gating are not suitable, and where it reduces liver dome motion. This results in only 20% of patients being treated with abdominal compression. For smaller centres, such a proportion of patients may be too small to justify the continued use of abdominal compression as staff skill and knowledge would be difficult to maintain.

### Practicalities

The workshop featured a session on the experiences of radiation oncology centres in NSW/ACT. This provided information on the full range of motion management techniques currently in use as well as the opportunity to discuss practical considerations such as the additional on-going cost or time required for particular motion management techniques, infection control considerations, contingency plans to allow patients to continue treatment in the case of breakdown and the potential burden of additional quality assurance requirements for new motion management equipment.

Survey C asked participants if any motion management techniques had once been used in their department but were now superseded. Abdominal compression, visual feedback and gating were all noted, with abdominal compression the most popular response (55%, 21 responses).

**Fig. 2:**
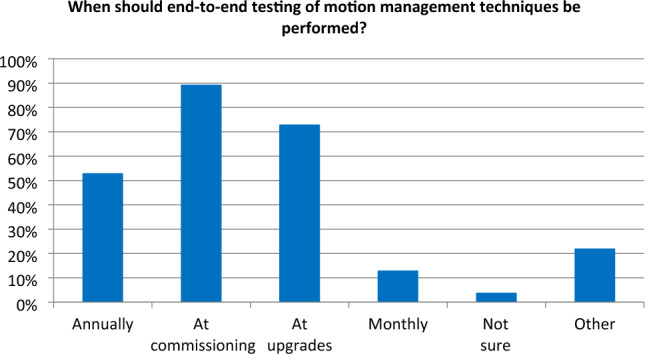
Opinions on the need for end-to-end testing of motion management techniques (55 responses)

The additional burden of quality assurance should be assessed against the value to the patient, and incorporated into staffing models. An example of this additional quality assurance is end-to-end testing of motion management systems. The majority of those surveyed felt this testing should be performed at commissioning, post-upgrade and annually (Fig. 2, Survey B).

### SBRT

With motion management an integral part of SBRT, many of the presentations and much of the discussion at the workshop addressed this treatment technique. Discussion was focused on two main areas, motion management for free-breathing patients and for patients treated in breath-hold with gating. Both techniques are commonly used, with 95% of centres using either a free-breathing gated technique or internal target volume (ITV)-based planning for lung SBRT patients and 70% of centres using a breath-hold technique for liver SBRT patients (Fig. 1, Survey A, as a percentage of centres treating with these techniques).

For both free-breathing and breath-hold treatments, workshop attendees were interested in which images should be used for the dose calculation. The advice from the workshop panelists aligns with previous published work, that small changes in the CT data set due to tumour position make little difference to the plan dosimetrically [18]. However, it is important to consider which image is more appropriate to be matched to the CBCT or 4DCBCT image on treatment. An example was provided for breath-hold treatments, where three or more breath-hold CT data sets may be used to create an ITV, acquired for the purpose of quantitating uncertainty in target position during breath-hold, with the most appropriate image the one showing the median tumour position. Another challenge identified at the workshop is matching of CBCT images to planning images. This can be difficult due to artifacts caused by fiducial markers or clips in the CBCT and differing methods of reconstruction and binning between the planning CT and the CBCT. The relevance of markers distant from the tumour was also questioned given recent publications [19].

### Free-breathing motion management

Motion management for free-breathing patients starts at simulation with acquisition of a 4DCT. Workshop participants were interested in the options available if a patient is breathing too slow, too fast or in an irregular way such that the image acquired are unreliable or technically can’t be acquired. Several options were discussed including coaching the patient to breath differently at simulation with the potential draw back that the patient’s breathing may not be the same at treatment. Monitoring breathing traces and using gating, either with a wide window around the patient’s normal breathing amplitude or focused around a part of the breathing cycle, were discussed as options that some centres have found help to ensure the target stays within the targeted region at treatment.

**Fig. 3:**
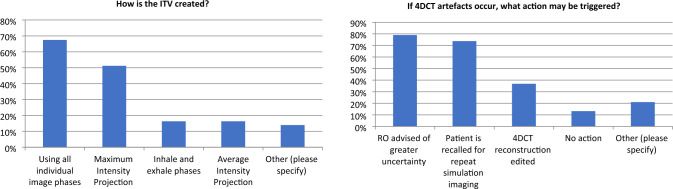
Survey A results showing how the ITV is created (left, 43 responses) and actions which may be triggered when an artifact occurs in a 4DCT image (right, 38 responses). Multiple responses were allowed for these questions

Practical issues associated with free breathing motion management were highlighted during the workshop. For example, that the tumour may not be completely visible on all phases of the 4DCT and that a review of the images is required to ensure the ITV is not compromised. This was also seen in Survey A where 98% of respondents agreed that 4DCT images require review due to the potential for compromised 4DCT imaging data (55 responses). A summary of approaches to manage artifacts in 4DCT images is presented in Fig. 3.

From Survey A, 93% of centres use 4DCT and 97% use ITV-based planning. Figure 3 shows the methods used to create an ITV, with most people using all individual image phases. The use of maximum intensity projection (MIP) CT to create an ITV was more popular in the AAPM survey [14], (73% AAPM and 51% this work). The delineation of targets using a MIP dataset has been shown to be faster [20] and result in a smaller target volume compared with contouring the tumour on all individual phases but it can also result in inadequately target coverage [20–25]. In addition to the planning images, workshop discussions indicated that, in addition to the simulation CT, diagnostic imaging may also be used as a source of information about tumour movement at a different time point.

### Emerging trends

The potential to reduce the irradiated normal tissue by using mid-ventilation based targets [26, 27] was presented at the workshop including practical steps towards implementation. Based on Survey A, this is yet to be implemented clinically in Australia and New Zealand.

New developments in motion management were discussed, including the potential for tumour tracking and dose-guided motion management to replace external surrogates and improve dosimetric accuracy. When asked about external surrogates, only 11% of survey respondents felt that these were sufficient for SBRT liver treatments compared to 78% for breast treatments, where the target is usually more superficial (Survey B, 55 responses).

**Table 2:** Responses to “Does your department use motion management for any of the following sites?” 66 responses recorded

Site	Responses (%)
Breast	94
Lung	91
Liver	56
Prostate	44
Pancreas	33
Kidney	30

## Discussion

The high attendance rate and range of presentations at the motion management workshop provided an opportunity for professionals to share practical experience and engage in collaborative learning without leaving their clinics. The high uptake of motion management, particularly for breast radiation therapy, and increasing interest in SGRT mean that additional sharing and learning opportunities may be required in the future. Organisers of future workshops should consider additional options to ensure all audience questions are addressed. These could include allocation of more time for questions, distribution of written responses after the workshop or facilitating private discussion between the attendees and presenters. Workshops could be viewed as a starting point for future work and discussion rather than a conclusion [28, 29].

One of the challenges identified by clinical physicists during the final session of the workshop was finding people with experience and knowledge. This workshop, and particularly the practical experience session, was designed to assist with and to facilitate knowledge-sharing. A physicist from a regional centre commented that ‘The forum has been a really good way to share and learn from other people’s experiences.’ A common theme was the need to adapt motion management approaches to the needs of individual patients. This may require new motion management options to become available. Increasing the range of techniques available to patients may present additional challenges in terms of training and resourcing.

Looking to the future, a minority of respondents felt that external surrogates were sufficient for SBRT liver (11%) with the remainder unsure or indicating that external surrogates were an inadequate surrogate for estimating internal motion. This may lead to the introduction or expansion of tumour and fiducial marker tracking more broadly.

Based on the workshop discussion and survey results, a move away from technologies that require expensive, non-environmentally friendly disposable items and particularly equipment that poses an infection risk [30] was identified. SGRT and x-ray based tumour tracking may have a role to play.

Despite the progress made in motion management, technological challenges remain: the quality of CBCT images for soft tissue structures was identified, as was the difficulty in acquiring adequate quality 4DCT images for some patients. Additionally, the fast pace of improvements in motion management also lead to training challenges and the risk that centres may not have the resources to implement and maintain a wide range of newly available technologies and techniques, particularly if patient numbers are small. Collaborative methods of working and learning may need to be explored to address these needs, with events such as this workshop to provide opportunities for sharing of knowledge, experience and ideas.

### Limitations of this work

A limitation of this work as a means to determine the current state-of-play in motion-management in Australasia is the reliance on workshop presentations, discussions, and surveys. These sources may introduce bias, as individuals with a particular interest in and knowledge of motion management are more inclined to participate in workshops and respond to surveys on this topic. While the survey response rate was high, obtaining current practice data through established reporting channels could enhance future research by mitigating response bias. For future work aiming to compare pre- and post-workshop opinions, the methodology could be improved by linking the two opinion surveys based on the respondent, focusing the survey questions, and encouraging the completion of the post-workshop opinions survey.

While this study presents the opinion of the participants, these opinions may not be linked to clinical outcomes data as such data may not exist. Linking technology to clinical outcomes is challenging due to the number of incremental changes that can occur over a protracted period. However, we suggest that the value of the workshop is in providing an opportunity to reflect on current practices, receive external peer review and develop a collaborative network to provide on-going peer review and support for the efficient and safe introduction of new technologies.

## Conclusion

Motion management is now seen as integral to the radiation therapy of many patients. The workshop presentations and the survey results show that motion management techniques for breast and lung radiation therapy are routinely incorporated as part of the standard of care in Australasia. However, further work is required to address the barriers to implementation and training which were highlighted during the workshop. Future trends in motion management may include an increase in the use of SGRT and the use of X-ray based monitoring and tracking where external surrogates are not sufficient to account for tumour motion. Additional training and collaboration opportunities may be required to address the needs of the radiation therapy community.

## Supplementary Information

Below is the link to the electronic supplementary material.

Supplementary material 1 (DOCX 22.8 kb)
