# Supplementary material for: Improving motion management in radiation therapy: findings from a workshop and survey in Australia and New Zealand
Source: Phys Eng Sci Med. 2024 May 28;47(3):813–20. doi: 10.1007/s13246-024-01405-0 (PMC11408578; doi:10.1007/s13246-024-01405-0)
Supplement: Supplementary file 1 — Supplementary material 1 (DOCX 22.8 kb) [file 13246_2024_1405_MOESM1_ESM.docx]

Appendix 1 – Survey Questions

**Survey A**

Q1: I have received the Participant Information Statement and email instructions.

Agree

Q2: Please select your profession from the drop-down box

ROMP, RT, RO, Other profession (please specify in comments), Other (please specify)

Q3: Which department/site do you work for? (if you work across multiple sites, please nominate the site where your provide the bulk of your services)* Please note - as described in the emailed participant information statement, this information will only be used for filtering data to ensure we have meaningful statistics on utilisation. All results presented and discussed will be de-identified and anonymity preserved.

Free Text Answer

Q4: Has someone from your site and in your profession previously completed this survey? If you are unsure, please click "No". This ensures we will get at least one respondent per site for Part 1 of this survey.

Yes - someone in my site & profession have completed this survey ---> You will be directed to Part 2 of this survey

No ---> You will be directed to Part 1 of this survey

Q5: Does your department use motion management for any of the following sites? (select all that apply)

No, none of the following sites, Lung, Liver, Pancreas, Prostate, Kidney, Breast, Other (please list)

Q6: Does your department use breath-hold and beam-gating techniques for breast patients (who are able to breath-hold)?

Left-Sided Breast, Right Sided Breast, Other (please specify)

Q7: If your department uses breath-hold gating (for sites other than breast) - how are margins determined?

N/A - not used in department, Standard for tumour type or site, Custom for patient (please give details e.g.based on repeat CTs, based on fluoro, adjusted after 1st fraction imaging...), Comments (e.g. other; extra information)

Q8: For the previously selected sites, which type of treatments do you use motion management for? (e.g. gating, breath-hold, 4DCT + motion based plans (ITV)...)

No, none of the following  sites, Lung, Liver, Pancreas, Prostate, Kidney, Breast, Other comments

Q9: For the sites where you previously indicated "SBRT Treatment only", is your department considering extending motion management strategies to conventional treatments as well?

No, none of the following  sites, Lung, Liver, Pancreas, Prostate, Kidney, Breast, Other comments

Q10: For the sites selected previously - indicate which (if any) of the following motion management techniques are currently used by the department for SBRT treatments.

No, none of the following  sites, Lung, Liver, Pancreas, Prostate, Kidney, Breast, Other comments

Breath-hold with gating, Free-breathing with gating (phase or "large gate window"), Abdominal Compression, Motion based plan (ITV, phase, mid-vent)

Q11: For the sites selected previously - indicate which (if any) of the following motion management techniques are currently used by the department for conventional treatments (non-SBRT)

No, none of the following  sites, Lung, Liver, Pancreas, Prostate, Kidney, Breast, Other comments

Breath-hold with gating, Free-breathing with gating (phase or "large gate window"), Abdominal Compression, Motion based plan (ITV, phase, mid-vent)

Q12: Does your department use Surface Guided Radiotherapy (SGRT) for motion management in any treatment sites (other than for cranial SRS)?

Yes, No, Procurement/implementation commenced, Under consideration, Other (please specify)

Q13: Does your department do screening of suitability of patient breathing, or pre-treatment assessment of target motion, before deciding on a motion management strategy?

Yes – both, Yes - only breathing ability, Yes - only target motion, No, Comments:

Q14 and 15: In your department, which methods are used to screen breathing suitability? (select all that apply) [The same question was asked twice but of different participants due to the survey logic.]

Respiratory function test, SGRT, ABC, RPM, Patient breathing ability, Target motion assessment, Other (please specify)

Q16: In your department, which methods are used to assess target motion (pre-treatment)? (select all that apply)

Planning images, Fluoroscopy, Other 4D imaging, Other (please specify)

Q17: Please indicate at which time points these screenings/assessments occur in your department? (select all that apply)

Patient breathing ability, Target motion assessment, Other (please specify)

Q18: Does your department use "coaching" or audio-visual (AV) feedback for patients where stable or reproducible breathing is required?

Yes - for free breathing treatments, Yes - for breath hold treatments, No, Sometimes (pleases detail), Comments

Q19: Does your department use ITV based planning for any sites?

Yes, No, Comments:

Q20: For ITV planning, how is the ITV created? (please specify in comments if site specific approaches are used)

Maximum Intensity Projection, Using all individual image phases, Average Intensity Projection, Inhale and exhale phases, Other (please specify)

Q21: Does your department use 4DCT at simulation for any sites?

Yes, No, Comments:

Q22: When 4DCT is used for planning, which image set is dose calculation done on?

Average 4DCT image, Free-breathing scan (additional scan), Other (please specify)

Q23: In your department, if 4DCT artefacts occur, what action may be triggered? (select all that apply)

Patient is recalled for repeat simulation imaging, 4DCT reconstruction edited, RO advised of greater uncertainty, No action, Other (please specify), Comments/Other (please specify)

Q24: Does your department do any monitoring of motion at the time of patient treatment?

Yes, No, Other (please specify)

Q25: For your SBRT treatments - how is motion monitored at treatment?

No, none of the following sites, Lung, Liver, Pancreas, Prostate, Kidney, Breast, Comments/Other (please specify):

Surrogate monitoring – e.g. RPM, ABC, SGRT, Imaging of seeds/markers, Imaging of target, Imaging of other anatomy, Other

Q26: For your conventional treatments (non-SBRT) - how is motion monitored at treatment?

No, none of the following sites, Lung, Liver, Pancreas, Prostate, Kidney, Breast, Comments/Other (please specify):

Surrogate monitoring – e.g. RPM, ABC, SGRT, Imaging of seeds/markers, Imaging of target, Imaging of other anatomy, Other

Q27: Does your department use intrafraction imaging during dose delivery (i.e. while the treatment beam is 'on')?

Yes, Selected sites only (please specify), No, Comments:

Q28: How are the intrafraction images used?

For monitoring only, With beam interlock, Manual beam-off, Comments:

Q29: For sites where you don't use intrafraction imaging during dose delivery, which methods/technologies does your department use to verify patient position and stability of target/s? (select all that apply)

ABC, RPM (or similar external markers), SGRT, Pre-treatment images (including mid-fraction), Post-treatment images, Other (please specify)

Q30: If your department uses post-treatment imaging and sees a deviation in patient position on these images, what actions are taken? (select any that may be used in your department)

Additional uncertainty reported to RO, Recalculation of dose at final position, Review of patient (e.g. re-screening, change technique), Review of monitoring system, Other (please specify)

Q31: If breathing motion at treatment is irregular or has changed enough to trigger a review/response, what actions may your department take? (select all that may be used)

Patient coached, Patient cancelled, Switch breathing technique, Change treatment/planning method (DCAT or 3DCRT), Increase margin, Other (please specify)

Q32: In your department, what kind of review process is used when assessing required breathing on treatment?

Formal documented decision tree/review protocol, Informal review of patient (e.g. based on experience/judgement), No review, Comments:

Q33: Does your department use any of the following monitoring technologies? (select all that apply)

ABC, RPM/RGSC, SGRT, 4DCBCT, 4DCT, Other (please specify)

Q34: For the motion monitoring technologies you have in department, what QA and QA schedule do you use?

ABC, RPM/RGSC, SGRT, 4DCBCT, 4DCT, [Insert text from Other], Comments:

Vendor/developer recommendations, International recommendations, In-house risk assessment, Other (please specify in comments), No regular QA performed

Q35: For the sites where you previously indicated no motion management is used, is your department considering implementing motion management?

No, none of the following sites, Lung, Liver, Pancreas, Prostate, Kidney, Breast, Other (please specify)

Yes, No, This site not treated in our department

Q36: Are there any motion management techniques that you once used but your department have now superseded? (e.g. Compression belts, double vac bags, fiducial markers..). If so, why?

Free Text Answer

Q37: Do you have any other comments or things you would like to say about motion management in your department?

Free Text Answer

**Survey B**

Q38: Do you consider that motion management techniques should be used for non-SBRT thoracic/abdominal patients?

Yes, No, Unsure, Comments:

Q39: How important do you consider performing patient breathing and/or target motion assessment at the following time points? Please indicate on the scale below.

Pre-simulation?, At simulation (or in post-sim assessment), Pre-treatment course starting, on machine (e.g. fraction 0)?, Other (please specify)

Completely unimportant, Not very important, Neutral, Important, Very Important

Q40: Should there be a lower threshold on motion before motion management is used?

Yes, No, Unsure, Comments:

Q41: Should there be an upper threshold of motion beyond which motion management must be used?

Yes, No, Unsure, Comments:

Q42: What QA/review should be required for patient 4DCT scans?

Review by ROMP, Review by RT/RO (referred to ROMP if issues), Unsure, None, Other (please specify)

Q43: Is external surrogate monitoring sufficient for treatment of the following sites/techniques?

DIBH Breast, SBRT Lung, SBRT Liver, Conventional fractionation thoracic/abdominal treatments, Comments

Yes, No, Unsure

Q44: When should end-to-end testing of motion management techniques be performed?

Annually, At commissioning, At upgrades, Monthly, Not sure, Other (please specify)

Q45: Should breath-hold patients also receive a free-breathing scan at their initial simulation to allow efficient transfer to a different (non-breath-hold) plan if breath holds are not successful or cannot be continued?

Yes, No Particular sites or circumstances (specify in comments), Unsure, Comments:

Q46: Do you think intrafraction monitoring during dose delivery (i.e. during 'beam on') should be a requirement for the following sites?

Lung, Liver, Pancreas, Prostate, Kidney, Breast, Other (please specify in comments), comments

Yes, No, Only for SBRT, Unsure

Q47: In your department, which technologies do you NOT have, but you think would be useful:

Abdominal Compression, SGRT, RPM/RGSC, ABC, Other (please specify in comments), Comments:

Q48: For each technology you identified, can you indicate the reason/s why do you not have it?

Abdominal Compression, SGRT, RPM/RGSC, ABC, Other (please specify in comments), Comments:

Have one on order, Seriously considering/business case prepared, Lacking in evidence for effectiveness, Cost, Lack of resources to implement (staff/time/knowledge), Lack of interest from key stakeholders

Q49: For each technology you identified, which clinical site/s do you think they would most benefit?

Abdominal Compression, SGRT, RPM/RGSC, ABC, Other (please specify in comments), Comments/Other (please specify)

Breast, Liver, Lung, Pancreas, Kidney, Other (give details in comments)

Q50: Are there any clinical sites to which your department does NOT apply motion management but you think should have motion management? Tick all that apply

Breast, Liver, Lung, Pancreas, Kidney, Prostate, Other (please specify)

Q51: Consider how motion management is used in your department, are there any clinical sites where you think you could change/modify your protocols to improve treatment quality

Breast, Liver, Lung, Pancreas, Kidney, Prostate, Other (please specify)

**Survey C**

Q1: I have received the Participant Information Statement and email instructions

Agree

Q2: Please select your profession from the drop-down box

ROMP, RT, RO, Other profession (please specify in comments), Other (please specify)

Q3: Did you complete the pre-workshop survey on your opinions about motion management?

Yes, No, Can't remember

Q4: Did you attend the "Motion Management in External Beam Radiotherapy" workshop

I attended all of the workshop, I attended most of the workshop, I attended parts of the workshop, I did not attend any of the workshop

Q5: In your department, which technologies do you NOT have, but you think would be useful:

Abdominal Compression, SGRT, RPM/RGSC, ABC, Other (please specify in comments), Comments:

Q6: For each technology you identified, which clinical site/s do you think they would most benefit?

Abdominal Compression, SGRT, RPM/RGSC, ABC, Other (please specify in comments), Comments/Other (please specify)

Q7: Are there any clinical sites to which your department does NOT apply motion management but you think should have motion management? Tick all that apply

Breast, Liver, Lung, Pancreas, Kidney, Prostate, Other (please specify)

Q8: Consider how motion management is used in your department, are there any clinical sites where you think you could change/modify your protocols to improve treatment quality Breast

Breast, Liver, Lung, Pancreas, Kidney, Prostate, Other (please specify)

Q9: As a result of attending the workshop, are there any technologies that you had not previously considered, but you now think might be useful:

Abdominal Compression, SGRT, RPM/RGSC, ABC, Other (please specify in comments), Comments:

Q10: For each technology you identified, which clinical site/s do you think they would most benefit? Abdominal Compression, SGRT, RPM/RGSC, ABC, Other (please specify in comments), Comments/Other (please specify)

Breast, Liver, Lung, Pancreas, Kidney, Prostate, Other (give details in comments)

Q11: As a result of attending the workshop, are there any clinical sites that you had not previously considered, which you now think would benefit from a motion management strategy? Tick all that apply

Breast, Liver, Lung, Pancreas, Kidney, Prostate, Other (please specify)

Q12: Consider how motion management is used in your department. As a result of attending the workshop, are there any clinical sites where you think you could change/modify your protocols to improve treatment quality?

Breast, Liver, Lung, Pancreas, Kidney, Prostate, Other (please specify)

Q13: Overall, based on your attendance at the workshop, have any of your opinions about motion management changed?

Yes, No, Comments/details:

Q14: How valuable did you find each of the workshop sessions? (please select N/A if you did not attend a session)(As a reminder, the program is here)

Day 1 Session 1: "Evolving Practice",

Day 1 Session 2: "Simulation & Planning",

Day 1 Session 3: "Surface Guided Monitoring",

Day 2 Session 1: "Imaging & Monitoring at treatment",

Day 2 Session 2: "Department Experiences",

Day 2 Session 2: "Trials, Development & the Future",

Comments:

Not at all valuable/ waste of time, Neutral, Somewhat valuable, Very valuable, Extremely valuable, N/A

Q15: What could be improved in future workshops?

Open-Ended Response

Q16: Do you consider that motion management techniques should be used for non-SBRT thoracic/abdominal patients?

Yes, No, Unsure, Comments:

Q17: How important do you consider performing patient breathing and/or target motion assessment at the following time points? Please indicate on the scale below. Pre-simulation?

At simulation (or in post-sim assessment), Pre-treatment course starting, on machine (e.g. fraction 0)?, Other (please specify)

Completely unimportant, Not very important, Neutral, Important, Very Important

Q18: Should there be a lower threshold on motion before motion management is used? Response

Yes, No, Unsure, Comments:

Q19: Should there be an upper threshold of motion beyond which motion management must be used?

Yes, No, Unsure, Other (please specify)

Q20: What QA/review should be required for patient 4DCT scans?

Review by ROMP, Review by RT/RO (referred to ROMP if issues), Unsure, None, Other (please specify)

Q21: Is external surrogate monitoring sufficient for treatment of the following sites/techniques?

DIBH Breast, SBRT Lung, SBRT Liver, Conventional fractionation thoracic/abdominal treatments, Comments

Yes, No, Unsure

Q22: When should end-to-end testing of motion management techniques be performed?

Annually, At commissioning, At upgrades, Monthly, Not sure, Other (please specify)

Q23: Should breath-hold patients also receive a free-breathing scan at their initial simulation to allow efficient transfer to a different (non-breath-hold) plan if breath holds are not successful or cannot be continued?

Yes, No, Particular sites or circumstances (specify in comments), Unsure, Comments:

Q24: Do you think intrafraction monitoring during dose delivery (i.e. during 'beam on') should be a requirement for the following sites?

Lung, Liver, Pancreas, Prostate, Kidney, Breast, Other (please specify in comments), comments
